# Multi-modal approach to preventing suicide in schools: a regionally-based UK pilot study

**DOI:** 10.3389/frcha.2026.1768052

**Published:** 2026-03-05

**Authors:** Emma Ashworth, Claire Hanlon, Molly McCarthy, Anna Hunt, Sio Wynne, Rio Foster, Jo Robinson, Samuel McKay, Pooja Saini

**Affiliations:** 1 School of Psychology, Liverpool John Moores University, Liverpool, United Kingdom; 2 Orygen Youth Health Research Centre, Centre for Youth Mental Health, University of Melbourne, Melbourne, VIC, Australia

**Keywords:** adolescents, mixed-methods, pilot study, school-based intervention, suicide prevention

## Abstract

**Background:**

Despite emerging evidence for the effectiveness of school-based suicide prevention programmes worldwide, there are few being implemented in the United Kingdom, and they have not been tested. Cultural transferability of school-based interventions cannot be guaranteed, and adaptations may be required. We aimed to conduct a pilot study of the Australian Multi-Modal Approach to Preventing Suicide in Schools (MAPSS) programme, to assess its feasibility and acceptability for delivery in the UK, and the potential for a future trial. MAPSS consists of three components: a universal workshop, screening to identify at-risk students, and a targeted intervention for students at-risk.

**Methods:**

A pilot study following a mixed-method explanatory design was conducted. A pre/post-test quantitative design was used with Year 10 students (aged 14-15 years) from two secondary schools in Northwest England (*N* = 417). Participants were assessed at three timepoints using online quantitative surveys. A qualitative process evaluation was conducted with 24 students and professionals.

**Results:**

MAPSS was generally considered to be acceptable and feasible, although there were issues with student engagement and logistics regarding delivery in schools. There were no issues with safety and missing data were within acceptable limits. Pre/post-test analyses indicated significant improvements in depression, hopelessness, and suicide literacy scores after the universal component. Pre/post-test analyses for at-risk students indicated that suicide ideation and quality of life scores were significantly higher after participating in the targeted component. Qualitative data demonstrated a strong perceived need for suicide prevention efforts in schools, with MAPSS thought to help increase awareness and identify students who were at-risk that schools had not previously been aware of.

**Conclusions:**

MAPSS is potentially appropriate to deliver in UK schools and may be beneficial for students, although requires further testing. A larger trial is considered feasible and is required to explore the utility and potential effectiveness of MAPSS. However, further work is needed to refine and adapt the intervention before a future trial can take place, with consideration of the logistical and staffing pressures within schools, and to facilitate student uptake and engagement.

## 1 Introduction

Suicide is the leading cause of death among children and young people (CYP) under 35 in the UK (1) and adolescent suicide rates in England and Wales are rising (2). Incidences of suicidal thoughts and behaviours occur with increasing frequency as young people reach adolescence (3), with 7.4% of 17-year-olds having a previous suicide attempt (4). In particular, Northwest England has experienced recent increases in attendances to Emergency Departments for CYP in suicidal crisis or for self-harm, with the number presenting significantly higher than the UK average (50). Suicidal ideation and behaviours are associated with a range of negative outcomes, including future death by suicide. Family and friends also experience negative effects, including significant distress, greater anxiety and depression, they are subsequently at an increased risk of attempting suicide themselves (5, 6). There is therefore an urgent need to develop and test acceptable and effective approaches to preventing suicide in this population in the UK.

Schools are considered an appropriate setting for the implementation of mental health prevention programmes, offering reach to many CYP, and have been identified as important locations for suicide prevention activities (7). A recent systematic review by Walsh et al. (8) identified 36 suicide prevention trials in secondary schools in the last 30 years, with meaningful reductions in suicidal thoughts and behaviours identified in approximately half of the trials. However, they noted that lower income countries and disadvantaged schools were underrepresented in the included studies, despite higher rates of suicidal behaviours, which may mean that findings are not representative. To date, the most commonly evaluated programmes include the Signs of Suicide (SOS) (9) intervention, Youth Aware of Mental Health (YAM) (10), and Question, Persuade and Refer (QPR) (8). In particular, YAM, a brief duration (five hours across four weeks) classroom-based psychoeducation programme, has gained traction in recent years across Europe. One large randomised controlled trial across 10 European countries identified significant reductions in the number of suicide attempts and severe suicidal ideation in adolescents at the 12-month follow-up stage (10). Similarly, a trial of the classroom-based SOS intervention in North American high schools (9, 11, 12) also evidenced reductions in suicide attempts at a three-month follow-up, although no significant effects were identified for suicidal ideation, and no longer-term follow-ups were conducted, meaning later attempts or deaths by suicide could not be recorded.

Currently, the majority of school suicide prevention programmes consist of a single intervention (e.g., a universal workshop); multi-modal approaches are less common and have not been as widely tested, despite a review by Robinson et al. (13) finding evidence to suggest that school-based psychoeducational interventions (i.e., universal approaches) coupled with screening have the potential to be effective (although higher-quality studies were needed to confirm this). This is in line with World Health Organisation (WHO) guidance (2018), which suggests that the most effective school-based suicide prevention interventions incorporate universal (delivered to a whole population), selective (for those with increased risk), and indicated (for those already experiencing suicidal thoughts or behaviours) approaches, in addition to general wellbeing promotion that can target related factors (8). Furthermore, it is thought that while school staff may be able to provide support to students, CYP are often reluctant to seek help from professionals, preferring informal sources of support (14). Thus, it is important that school-based prevention efforts not only target school staff, but also fellow students.

The Mult-Modal Approach to Preventing Suicide in Schools (MAPSS) programme is one intervention that takes all of these aforementioned issues into consideration. Unlike other school-based interventions, MAPSS involves a tiered approach, with the aim of promoting knowledge, awareness, and help-seeking through a universal workshop (safeTALK), in order to help CYP keep themselves and their peers safe. CYP are subsequently screened for suicide risk, with additional support then provided to CYP identified as at-risk of suicide through an online cognitive behavioural therapy programme (Reframe-IT), aiming to equip them with the skills to reframe negative thought processes, enhance coping skills, and develop safety plans (see methods for further details). This structure reflects WHO (15) recommendations for public health and suicide prevention and aims to provide a comprehensive, tiered response across the continuum of suicide risk in schools.

MAPSS was developed and is currently being trialled in secondary schools in Victoria, Australia (16), following successful pilot studies indicating that the interventions were safe and acceptable for use with school students. In particular, safeTALK demonstrated increased knowledge about suicide, confidence to intervene, willingness to talk about suicide, and help-seeking intentions for suicidal thoughts (17), with no iatrogenic effects reported. Reframe-IT demonstrated effects on suicidal ideation and depressive symptoms (5, 18, 19). Reframe-IT has also shown positive effects in international adaptations [e.g., in Chile; (20)]. Despite showing promise, MAPSS has not been widely implemented, and has not been previously tested in the UK; an issue we seek to address.

However, the cultural transferability of MAPSS from Australia to the UK cannot be assumed. Interventions that have worked in one setting or context often do not work across other settings, particularly when delivered in schools (21). Indeed, Wigelsworth et al. (22) found that school-based interventions implemented within the same country in which they were developed showed greater effects than those transported abroad. Variations in the transferability of school-based interventions are thought to be due to a wide range of contextual and cultural factors/differences, with adaptations often needed before an intervention can be successfully imported (23). Further, if an intervention does not have high social validity, meaning it is not viewed as acceptable, useful, and feasible by intervention deliverers (e.g., school staff) and/or recipients (e.g., students), then it is likely to fail (24, 25). While MAPSS has been found to be acceptable in Australia, its acceptability in the UK is not yet known. Although the UK and Australian education systems appear broadly similar (e.g., similar education stages and methods of delivery), schools in Victoria typically have more professional mental health support readily available (e.g., in Victoria, the “Mental Health Practitioners in Schools” programme ensures every secondary school is funded to employ at least one dedicated mental health professional, with funding also available for school to access a “menu” of mental health support (26). In the UK, mental health provision in schools is not standardised, and schools often have to refer students to external support, with long waiting times common (27). A range of other broader cultural factors (e.g., levels of stigma, public perception, availability of healthcare services) may also play a role. Thus, the transferability of MAPSS is not guaranteed and pilot work is needed to understand factors that may influence its implementation and acceptability, as well as any adaptations that are required before it can be delivered in the UK (22).

Our recent scoping study (7) ascertained the views of CYP, parents, and professionals regarding the potential acceptability of MAPSS for the UK. All participants advocated the importance of MAPSS and suggested some adaptations to the Australian intervention. An adapted version of MAPSS has since been co-developed with CYP and professionals. Adaptations were largely focused on Reframe-IT, with an updated version being produced, named Reframe IT-UK. Changes to Reframe IT-UK were generally surface-level, such as using British actors for videos, modernising content (e.g., discussions of COVID-19 and removal of references to outdated technology), providing UK sources of support, and making completion of a safety plan compulsory at the beginning of the programme (previously an optional module). safeTALK content was adapted to reflect UK statistics and services, and to allow it to fit into a UK school timetable. The screening strategy remained unchanged. This adapted version of MAPSS now needs piloting in UK schools, before it can be delivered and trialled at-scale. Its social validity, feasibility, and safety should be established, along with any further necessary cultural or contextual adaptations, to help ensure success.

### 1.1 Aims

We aimed to conduct a small six-month pilot study of MAPSS in two secondary schools in Northwest England. The study assessed the feasibility and acceptability of the adapted version of MAPSS for delivery in the UK, as well as the feasibility of a larger trial. Specifically, we aimed to test:

1. The acceptability and social validity of safeTALK, screening, and Reframe IT-UK,
2. The safety of MAPSS for delivery in UK schools,
3. The feasibility of conducting a larger trial of MAPSS, operationalised as the proportion of missing data on completed assessment, change or variability on a range of suicide ideation, mental health, and wellbeing outcome measures, the extent to which schools delivered MAPSS as intended, and the extent to which young people engaged in MAPSS.

## 2 Methods

### 2.1 Design

**Figure 1:**
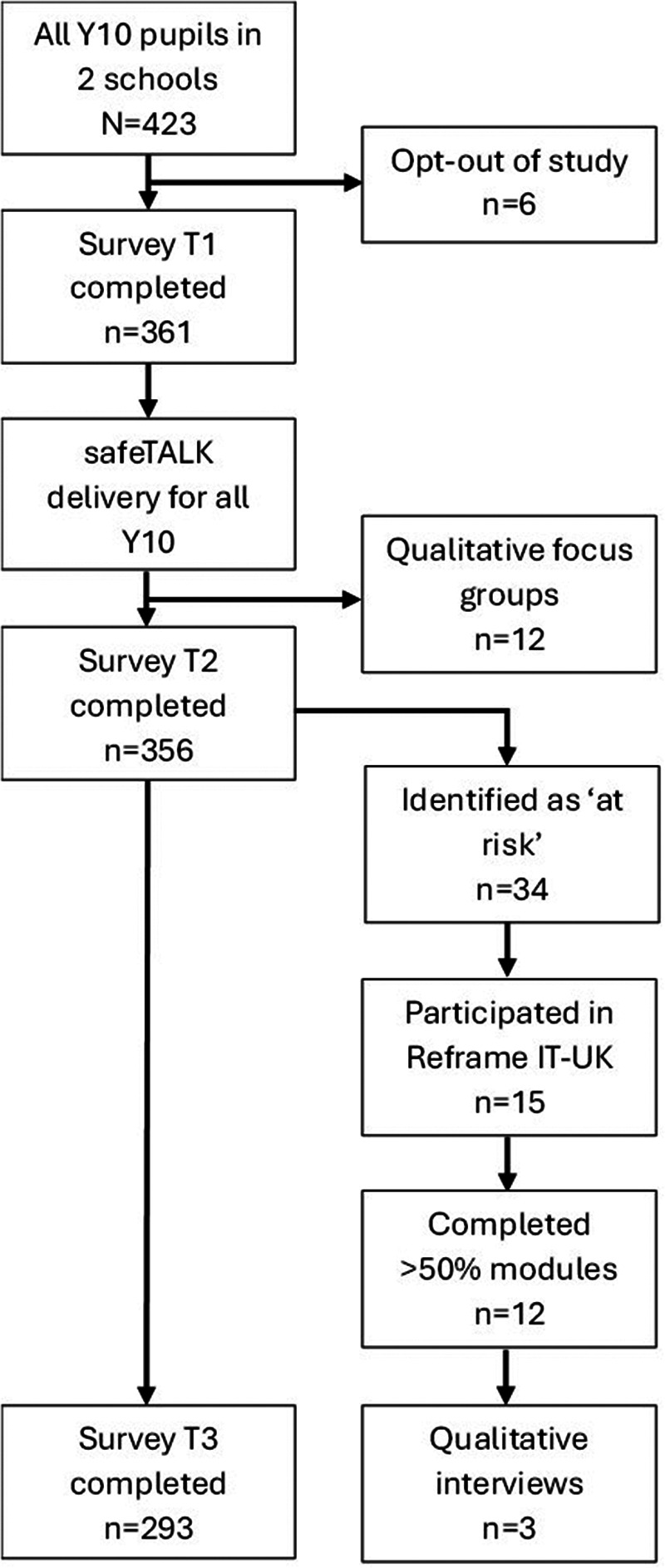
Overview of study process.

A small pilot study of MAPSS was conducted, beginning February 2023, employing a mixed-methods sequential explanatory (QUANTqual) design (28), with qualitative data providing a complementary role to help explain quantitative findings and to provide further contextual information. For the quantitative element, a pre/post-test design was used. Participants were assessed using quantitative online surveys immediately before the programme (baseline; T1), two-three weeks after the universal workshop (T2), and after completion of the indicated component (T3). A qualitative process evaluation was also conducted to explore social validity, acceptability, and feasibility, and to examine factors affecting implementation. Participants' involvement in each phase of the study is illustrated in Figure 1. Ethical approval was granted via the authors' University Research Ethics Committee (ref: 23/PSY/003) and the Consolidated Criteria for Reporting Qualitative Research (COREQ, see Supplementary Files) was utilised when describing qualitative methods and findings.

### 2.2 Participants and recruitment

Secondary schools in Northwest England were recruited via convenience sampling, through existing contacts. School gatekeepers (i.e., a member of the school's senior leadership team), were provided with a participant information sheet and were asked to sign and return an opt-in consent form. Two state secondary schools were recruited, one co-educational and one single-sex (male) selective grammar school.

#### 2.2.1 Quantitative strand

All Year 10 (Y10) students (aged 14-15 years) in participating schools were invited to take part. Gatekeepers were asked to provide parents/carers of all students with a participant information sheet and opt-out consent form. All students with parental consent were asked to provide opt-in assent at the beginning of the surveys at each timepoint. 417 students consented and participated in the quantitative data collection; 259 from the first school and 158 from the second. Four parents did not consent in school 1, and two parents did not consent in school 2. All young people assented, although some did not complete all of the surveys.

72% (*N* = 302) identified as a boy/man, 3.1% (*N* = 13) indicated that they had previously received a diagnosis for depression, 7.2% (*N* = 30) for anxiety, 3.6% (*N* = 15) for ADHD, and 2.2% (*N* = 9) for autism. Further demographic details are provided in Table 1.

**Table 1:** Participant demographics overall and by school.

Demographic characteristic		Total	School 1	School 2
Participants (*N*)		417	259	158
Age	14	37.2% (*n* = 155)	38.6% (*n* = 100)	34.8% (*n* = 55)
	15	61.4% (*n* = 256)	60.2% (*n* = 152)	63.3% (*n* = 100)
	16	1.0% (*n* = 4)	0.8% (*n* = 2)	1.3% (*n* = 2)
Gender	Boy/man	72.4% (*n* = 302)	57.5% (*n* = 149)	96.8% (*n* = 153)
	Girl/woman	22.1% (*n* = 92)	35.1% (*n* = 91)	0.6% (*n* = 1)
	Non-binary	0.7% (*n* = 3)	1.2% (*n* = 3)	–
	Transgender	1.7% (*n* = 7)	2.3% (*n* = 6)	0.6% (*n* = 1)
	Prefer not to say	1.9% (*n* = 8)	2.3% (*n* = 6)	1.3% (*n* = 2)
	Other	1.2% (*n* = 5)	1.5% (*n* = 4)	0.6% (*n* = 1)

34 CYP (26 in school 1; 8 in school 2) students (8%) were identified as at-risk and therefore eligible to participate in Reframe IT-UK. 15 of those opted to participate in the programme (including both parents' and students' consent).

#### 2.2.2 Qualitative strand

Participants included students, school staff and gatekeepers, and external workshop facilitators. Professional participants were provided with a participant information sheet and opt-in consent form by the research team for one-to-one interviews. For students, gatekeepers were asked to identify students who may be interested in taking part. They were advised to recruit students who they felt had an appropriate level of maturity and who were unlikely to find the research distressing. While this may have introduced some recruitment bias, it was a necessary requirement for ethical and safety purposes. Gatekeepers then obtained written opt-in consent from parents. Next, the researcher visited the school and provided students with a participant information sheet and opt-in assent form. Students took part in small focus groups regarding the psychoeducation workshop at T2 (to reduce power imbalances and ease nerves), and one-to-one interviews at T3, for those had taken part in Reframe IT-UK (due to the sensitive and personal nature of intervention participation). Workshop facilitators were approached via their own organisation and invited to take part in a one-to-one interview.

Five school staff members took part in one-to-one interviews, and 15 students participated across three interviews and two focus groups. Further details are provided in Table 2. Four workshop facilitators also took part in one-to-one interviews; all were female and worked for UK-based suicide prevention charities.

**Table 2:** Qualitative participants across schools.

School 1			School 2		
Identifier	Role	Timepoint	Identifier	Role	Timepoint
School staff (SS)					
Staff 1	Cover supervisor (staff member managing classrooms during teacher absences) and member of safeguarding team (staff member with responsibility for ensuring student safety)	T2	Staff 4	Learning mentor (non-teaching staff who support students’ wellbeing and barriers to learning)	T2
Staff 2	Middle leadership and class teacher	T2	Staff 5	Middle leadership, class teacher, and gatekeeper	T3
Staff 3	Assistant headteacher, designated safeguarding lead (with school-level responsibility for student safety), gatekeeper	T3			
Children and young people (CYP)					
Focus Group 1	Seven students who had participated in the workshop	T2	Focus Group 2	Five students who had participated in the workshop	T2
Student 1	Reframe IT-UK participant	T3			
Student 2	Reframe IT-UK participant	T3			
Student 3	Reframe IT-UK participant	T3			

### 2.3 Intervention delivery and safety mechanisms

MAPSS consists of multiple different components (see Figure 1):

1. A universal psychoeducation session, safeTALK. safeTALK was developed by LivingWorks Education, designed to be suitable for anyone over the age of 14. Following baseline survey completion (T1), safeTALK was delivered as a single 3.5-hour face-to-face workshop for all students in Year 10, in classroom-sized groups of students, by external facilitators from two suicide prevention organisations/charities (both followed the safeTALK model and were accredited by LivingWorks). safeTALK is an evidence-based programme with content that includes helping participants understand suicide warning signs in themselves and others, gaining knowledge about sources of support, applying basic “TALK” steps (Tell, Ask, Listen, and KeepSafe), and understanding how to signpost peers to appropriate services/support. This component is grounded in theoretical models suggesting that increasing suicide-related knowledge, improving attitudes, and enhancing self-efficacy to intervene are predictive of bystander interventions (17). safeTALK workshops are designed to be interactive, with videos, scenarios, activities, group discussions, and role play included. Schools were asked to ensure an adequate space was available, where students could move around, and to make technology available for the facilitators to share media. A member of school staff was present in case of any behavioural or wellbeing needs.
2. Screening. To identify at-risk students, screening was implemented using self-report measures after safeTALK delivery, as part of the T2 surveys. This component is based on three core assumptions: 1) suicidal thoughts are prevalent among adolescents but frequently go undetected by existing support systems, 2) direct inquiry about suicide increases identification and opportunity for timely intervention, and 3) asking about suicide does not elevate risk. Students who reported suicidal ideation within the past four weeks [Suicide Ideation Attributes Scale (SIDAS) score of 21 or higher, see below for further details (29)], or any level of current suicidal ideation (single multiple-choice item) were considered to be “at-risk”. Results of the screening were reviewed the same day by the research team, and CYP who were deemed to be at-risk were referred back to the school for support. The study safety protocol stipulated that screening was not completed on Fridays or before school holidays, to ensure participants received follow-up support promptly.
  Schools agreed at the beginning of the study to acknowledge receipt of the names of at-risk CYP and provide them with support within 24 h. It was explicitly agreed with schools that while it was the responsibility of the research team to provide them with names of students who were at-risk, the schools continued to hold full responsibility for students' safety at all times beyond that point, and they were required to adhere to their usual safeguarding policy and procedures throughout (i.e., the steps they would ordinarily follow if a student disclosed thoughts of suicide in school, including speaking with the student, contacting parents, triaging and referring to external agencies for support as necessary).
3. An online suicide-specific cognitive behavioural therapy (CBT) based intervention, Reframe IT-UK. This was designed for at-risk young people, and originally developed in Australia (5, 18). Schools were advised to offer all CYP identified as at-risk in the screening to take part in Reframe IT-UK. Reframe-IT is designed to reduce suicidal ideation in adolescents by targeting maladaptive thoughts and hopelessness through eight 20-minute online modules focused on cognitive restructuring, behavioural activation, problem-solving, and emotion regulation. The core assumption of Reframe-IT is that structured CBT skills can help adolescents manage distress and reduce suicidal thinking. Digital delivery also increases accessibility and reduces stigma, making it well-suited for school settings. An adult “host” character guides the user through the modules and activities. Each module contains video diaries, following the lives of two “characters” experiencing suicidal thoughts, along with two activities based on standard CBT exercises. Reframe IT-UK also has a message board through which participants can communicate with a moderator, a mood diary function, a mood “check-in” at the beginning and end of every module to ascertain current levels of suicide ideation, and a series of factsheets and information on local and national helplines and services. The website also includes a compulsory interactive safety plan, a tab with information about getting help locally/nationally, and an emergency help page.
  Participating students were provided with a login for the website and progressed sequentially through the content. They completed modules individually during school time, supported on a one-to-one basis by a staff member with responsibility for pastoral care/student wellbeing. Schools were asked to be discreet when taking students out of lessons for Reframe IT-UK, and were advised to discuss with the student how they would like this to happen (e.g., what they would like their classmates to be told). Modules unlocked once per week, during the daytime, when the preceding module was complete. This was so that modules could be completed during the school day, and to prevent CYP completing the modules in their own time when support may not be available. Modules were also blocked from unlocking during school holidays. However, once the module had been completed, CYP could review its contents again at any time. They could also access the help pages and their safety plan whenever they needed.
  Responses to the mood checkers and diaries were reviewed once every 24 h (during term time) by an administrator (a member of the research team), and the names of any students indicating distress or thoughts of suicide were provided to the school. If a student posted a message that concerned an administrator, they would reply with information about support services (linked on the Reframe IT-UK platform) and would advise the participant that they were passing their details onto their school, so that they could receive further support. As above, the schools were asked to support them in accordance with their safeguarding policy. During school holidays, a message was posted on the message board, advising CYP that any posts would not be read and signposting them to other avenues of support.
4. Training for school staff and parents/carers. This was delivered by professionals from an external suicide prevention organisation (Papyrus UK, a national suicide prevention charity for young people) at T1. It was designed to provide staff and parents with the skills they need to support CYP who are identified as at-risk during the programme, ensuring schools were adequately equipped to deliver the intervention safely and manage any disclosures.
  A minimum of six staff from each school received “Suicide Prevention – Overview Tutorial” (SP-OT). SP-OT is a single session lasting 1.5 h online. Content includes recognising warning signs in CYP, appropriate language to use, strategies to keep CYP safe, and where and how to seek further support. It was the schools' decision as to which staff members attended the training; however staff involved typically included members of schools' senior leadership teams, such as safeguarding leads (schools in the UK are legally required to name a senior member of staff with lead responsibility for safeguarding and child protection) and pastoral leads (i.e., those with school-level responsibility for students' wellbeing), as well as staff who were Heads of Year (with responsibility for managing each Year group as a whole), and staff who delivered mental health and wellbeing support. Parents of all Year 10 students were offered “Suicide Prevention - Awareness, Resource, Knowledge” (SP-ARK), a 30-minute training session online, along with support packs, to help equip them with awareness of warning signs, and knowledge of how and where to seek help.

### 2.4 Measures

#### 2.4.1 Quantitative strand

##### 2.4.1.1 Demographics

Demographic information (age, gender, diagnosed mental health conditions, and neurodiversity) were collected from participants as part of the online surveys at T1.

##### 2.4.1.2 Acceptability and social validity

Social validity (i.e., perceptions of acceptability, feasibility, and utility) of safeTALK was assessed at T2 using purpose-designed items. Participants' views on Reframe IT-UK were assessed using purpose-designed items at T3. Participants were asked to rate the extent (not at all/somewhat/very) to which they found the components interesting and useful, and whether they would recommend them to other young people (yes/no). Reframe IT-UK participants were also asked to rate each module on a five-point Likert scale (Strongly disliked-really liked).

##### 2.4.1.3 Safety

Participants were asked to rate the extent (not at all/somewhat/very) to which they found the workshops and Reframe IT-UK components upsetting, at T2 and T3 respectively. Safety was also assessed as part of routine monitoring of Adverse and Serious Adverse Events (AEs; SAEs), following NIHR guidance (30). Any unfavourable and unintended clinical sign, symptom, or observation temporally associated with the use of an intervention were considered as AEs. AEs that resulted in hospitalisation for any reason, life-threatening situations (including a suicide attempt), or death were regarded as SAEs. Schools were required to inform the research team of any AEs or SAEs that occurred during the course of the study, and these would be logged in a study database. The causality of AEs and SAEs (i.e., their relationship to intervention treatment) would then be assessed by a suitably qualified study team member, to determine the plausibility of the SAE being due to the intervention. Any SAE would be reported to the funder and to the relevant ethics committees within 24 h of the research team becoming aware of its occurrence, where a decision would be made about next steps.

##### 2.4.1.4 Feasibility of the trial

Data were collected on: 1) the missing data on completed assessment; 2) change or variability on secondary outcome measures (see below); and, 3) the extent to which schools implemented and supported the accessibility of MAPSS (a single-item question at T2 asked students if they had completed all/some/none of safeTALK; dosage of and engagement with Reframe IT-UK were extracted from the programme website).

##### 2.4.1.5 Secondary outcomes

1. Change in past four-week suicidal ideation, assessed via the SIDAS (29) at T2 and T3. The SIDAS is a self-report measure designed for web-based use to screen individuals in the community for presence and severity of suicidal thoughts. It comprises five items, each targeting an attribute of suicidal thoughts: frequency, controllability, closeness to attempt, level of distress, and impact on daily functioning. Responses are measured on a 10-point scale. Total SIDAS scores are calculated as the sum of the five items, with controllability reverse scored. A higher total score reflects more severe suicidal thoughts (range = 0–50). Scores of 21 or over are considered to indicate significant suicide ideation; this was used to help determine eligibility for Reframe IT-UK. The SIDAS has demonstrated high internal consistency and good convergent validity (*α* = 0.91) (29) previously, although reliability was poor in the present sample (*α* = 0.55).
2. Change in symptoms of depression, assessed using the Patient Health Questionnaire – 9 item version (PHQ-9) (31) at all timepoints. Participants are asked to indicate how often they have been bothered by nine problems over the past two weeks on a four-point Likert scale. Scores are summed, with higher scores indicative of greater distress (range = 0–27). Total scores of 5, 10, 15, and 20 represent cut-points for mild, moderate, moderately severe and severe depression, respectively. The final question of the PHQ-9 asks participants to indicate the number of days they have had thoughts that they would be better off dead, or hurting themselves in some way. Responses on this item were used to help determine eligibility for Reframe IT-UK. The PHQ-9 has been validated for use with adolescents, with good psychometric properties and high internal consistency, both previously (*α* = 0.86) (32), and in the current sample (*α* = 0.86).
3. Changes in hopelessness, assessed using the Brief-H-Pos, a two-item positively worded measure of hopelessness (33) at all timepoints. Respondents indicate agreement on a five-point scale (range = 2–10), with higher scores indicating higher hopelessness. The Brief-H-Pos has been adapted from the Beck Hopelessness Scale, which has been used extensively with adolescents. Its psychometric properties are satisfactory, with good internal consistency (*α* = 0.77) and concurrent validity with the Beck Hopelessness Scale (33), both previously and in the present sample (*α* = 0.70).
4. Changes in intentions to seek help, assessed using part two of the General Help Seeking Questionnaire (GHSQ) (34) at all timepoints. The GHSQ presents participants with a list of potential sources of help and asks them to indicate the likelihood that they would approach that source if they were experiencing suicidal thoughts, on a five-point scale. Higher scores indicate greater levels of intended help-seeking (range = 0–50). The GHSQ has been validated for use in high school students, with satisfactory reliability and validity (*α* = 0.83; test-retest = 0.88) (34). Internal consistency was good in the present sample (*α* = 0.77).
5. Change in health-related quality of life, assessed using the Child Health Utility–9 (CHU9D) (35) at all timepoints. The CHU9D can be used to derive quality-adjusted life years. The CHU9D is a nine-item multi-attribute utility instrument, with five response levels. Higher sores indicate greater behavioural or emotional difficulties (range = 9-45). The CHU9D was developed for use with CYP and has demonstrated good internal consistency (*α*>0.7) and strong test-retest reliability [r > 0.6; (36)], both previously and in the present sample (*α*=0.87).
6. Change in suicide literacy, assessed using an adapted version of the Literacy of Suicide Scale (LOSS) (37) at all timepoints. The LOSS contains 12 statements rated on a “true/false/don't know” scale. The scale provides a total literacy score (percent correct) where higher scores indicate greater suicide literacy.

#### 2.4.2 Qualitative strand

Bespoke interview schedules were produced for each participant group and each timepoint, but all explored: 1) social validity of MAPSS; and 2) *how* MAPSS was implemented and *why* it was implemented in this way. Interview schedules focused on the core dimensions of implementation (e.g., fidelity, dosage, quality, participant responsiveness) (38). A range of factors that may have affected implementation at the different domains/levels were also explored (38, 39). Overarching this was Wolf's social validity framework (1978). Interview schedules were semi-structured, designed to act as a guide to ensure specific topics were addressed whilst also allowing for unanticipated responses (40).

### 2.5 Procedure

#### 2.5.1 Quantitative strand

Following consent, schools arranged a suitable time for students to complete baseline (T1) surveys online. Two members of the research team (CH, EA, or PS) visited the schools and provided students with a brief presentation, outlining the purpose of the study, what was involved, and reminding them of their rights, in line with Demkowicz et al.'s good practice guidance (47). Students were then provided with the survey link (via QuestionPro) and were asked to tick a box to indicate assent. If any students did not want to take part, schools were asked to provide them with an alternative activity. For safety reasons, the researchers reviewed all students' responses regarding suicide ideation on the same day as the screening was completed. Researchers then provided safeguarding leads with the name of any at-risk students, asking them to follow-up with the students within 24 h, following their school's safeguarding policy. This procedure was repeated at T2 and T3.

Between T1 and T2 data collection, schools received safeTALK. At T2, any students scoring in the at-risk range on the screening were approached by the gatekeeper and offered Reframe IT-UK. Students receiving Reframe IT-UK then completed the eight modules in the 10 weeks between T2 and T3 (i.e., approximately one module per week, while accounting for disruptions such as school holidays). Completion of Reframe IT-UK took place during school time, and a pastoral member of staff supported students while they completed the programme.

#### 2.5.2 Qualitative strand

Qualitative fieldwork visits were conducted at T2 and T3 to interview staff and students (detailed in Table 2). School staff and workshop facilitator interviews took place via Microsoft Teams or in a private office. All CYP interviews and focus groups took place in quiet, private rooms in school, during the school day. Interviews were conducted by two researchers (EA and CH), the Principal Investigator and the research assistant for the study. Both are educated to doctoral level, have extensive experience working with CYP within and outside of research settings, and have received additional training in qualitative research methods and safeguarding. Interviews and focus groups lasted between 18 and 40 min for school staff and students (average = 27 and 28 min respectively) and between 32 and 54 min for safeTALK facilitators (average = 41 min). Focus groups comprised three to seven students. Encrypted Dictaphones or the Microsoft Teams transcription feature were used to produce transcripts, which were then anonymised.

### 2.6 Analytic strategy

#### 2.6.1 Quantitative strand

Data were analysed in IBM SPSS 27. Descriptive statistics were conducted for all measures on data collected across the two schools. However, as the universal workshop (safeTALK) was delivered by different organisations across the two schools, we also chose to examine social validity measures within each school individually, to explore whether acceptability was influenced by any potential variability in delivery. Where appropriate, paired samples t-tests were conducted to examine changes in secondary outcomes across timepoints. Paired samples t-tests were used as part of the pre/post-test design, to identify changes from baseline for all students immediately after the workshop (T2), and to identify if these changes were sustained at follow-up (T3). They were also used with students who participated in Reframe IT-UK, to identify changes in students before (T2) and after (T3) receiving the targeted intervention.

#### 2.6.2 Qualitative strand

The audio recordings were transcribed verbatim using the Otter programme (http://www.otter.ai) or Microsoft Teams feature and were reviewed and validated by the researchers. Framework analysis was used to analyse the data in NVivo 11, following Ritchie and Spencer's (41) five stages of thematic framework analysis. To start, two members of the research team (MM, RF) familiarised themselves with the dataset. Following discussions with other team members (EA, PS, CH), initial themes and codes were generated to produce a thematic framework. Both an inductive and deductive approach was used, in line with Ritche and Spencer's (41) approach. The remaining transcripts were then coded against this framework, and themes were refined. An iterative process was utilised, which enabled the continual revision of the thematic framework until final major themes were agreed by the team, ensuring trustworthiness and rigour. Once the final framework was agreed, each theme was defined and interpreted.

##### 2.6.2.1 Reflexivity

The interviews and focus groups were conducted between participants and the UK MAPSS research team, who were independent to and unaffiliated with the schools, and were independent evaluators of MAPSS. This was articulated to participants to help ensure they felt confident to share their experiences and opinions, both positive and negative. We also emphasised at the outset of the interviews and focus groups, and in study information sheets, that these were confidential conversations between staff and the research team and that individuals or their schools would not be identified in any report of the findings. We also clarified that we had not been involved in the development of the interventions and that we were interested in a range of perspectives. This perspective was carried into our analysis, where we remained open to the possibility that MAPSS may not be the best fit for English schools, for certain students, or that adaptations may be needed. The MAPSS developers were not involved in the data collection or analysis procedures.

However, it is possible that the development of our interview schedule and our approach to data analysis may have been influenced by our own prior experience and knowledge as researchers within the field. We have a range of personal and professional experience in suicide prevention, and delivering and evaluating various school-based mental health interventions, which may have influenced the approach we took to data collection and analysis. We therefore sought to ensure that we took an open, enquiring stance throughout the interviews, asking non-leading questions about participants' experiences and opinions. We also sought to ensure we gathered balanced perspectives, focusing on both barriers and facilitators, things that worked well or less well, and suggestions for improvements. Our analysis was inductive and regular reflexive discussions were held between the analysts and the rest of the research team to ensure balance.

## 3 Results

### 3.1 Acceptability and social validity

Of those who answered the safeTALK acceptability questions (*N* = 261), the majority reported that it was “somewhat” or “very” interesting' (79.7%; *n* = 204) and “somewhat” or “very” useful (83.2%; *n* = 213). However, results varied considerably between the two schools, with a higher proportion of students in the first school indicating that it was “not at all” interesting (28.5%) and “not at all” useful (23.8%) compared to the second school (7.6% and 5.7% respectively). 81.0% (*n* = 205) would recommend safeTALK to other young people, although again this varied between the two schools (75.0% in school 1 vs. 89.5% in school 2).

Ten students indicated that they had been offered Reframe IT-UK, although only nine responded to the follow-up questions. When asked whether they felt the programme was useful, the most common response was “neither agree nor disagree” (*n* = 5; 55.6%), while two participants disagreed (22.2%) and two agreed (22.2%). Five participants said they would recommend Reframe IT-UK to a friend, two said they would not, and the remainder did not answer the question. One participant indicated that they used Reframe IT-UK outside of the school sessions. Participants were also asked their opinions on individual elements of the programme; responses are provided in the Supplementary Table S1.

### 3.2 Safety

The majority (93.3%) of students across both schools did not find the workshop upsetting, and 100% of Reframe IT-UK respondents indicated that Reframe IT-UK was not upsetting. No adverse events or serious adverse events relating to the study were reported by the schools to the research team over the course of the study.

### 3.3 Trial feasibility

#### 3.3.1 Missing data

417 CYP received consent to take part. Of those, 86.6% (*n* = 361) took part in the surveys at T1, 85.4% took part at T2 (*n* = 356), and 70.3% took part at T3 (*n* = 293).

#### 3.3.2 safeTALK delivery

78.2% (*n* = 241) of those who responded to the question at T2 indicated that they took part in safeTALK, while 6.5% (*n* = 20) indicated that they took part but arrived late or left early. 14.9% (*N* = 46) said that they did not take part. Data were missing for 109 participants.

#### 3.3.3 Reframe IT-UK implementation

Data were extracted from the Reframe IT-UK website regarding dosage (i.e., number of modules completed) and engagement (i.e., number of posts on the message board or mood diary, number of mood check-ins completed). Of the 34 students identified as “at-risk” and eligible for Reframe IT-UK, 15 decided to participate and were registered on the online platform. 23% (*n* = 3) completed all eight modules of Reframe IT-UK, 69% (*n* = 9) completed five or more modules, and 23% (*n* = 3) did not complete any modules. The number of modules completed is illustrated in the Supplementary Figure S1.

Of those who used Reframe IT-UK (*n* = 12), 41.7% (*n* = 5) posted on the message board, with the number of messages ranging from one (*n* = 1) to five (*n* = 1). 25% (*n* = 3) used the mood diary; two participants used it once, and one participant used it five times. Only two participants completed all 16 mood check-ins, seven completed between two and 14, and the remainder did not complete any. A scatterplot is provided in the Supplementary Figure S2, providing an overview of suicide ideation scores at each check-in.

#### 3.3.4 Secondary outcomes

##### 3.3.4.1 SafeTALK

Table 3 presents the mean scores for each of the secondary outcome measures, for each timepoint. In terms of pre- and post-safeTALK (T1 vs. T2), average depression scores fell from 5.67 to 5.34, and hopelessness scores fell from 4.00 to 3.69. Average help-seeking intentions rose from 22.08 to 23.12, and suicide literacy rose from 6.25 to 6.44. Paired t-tests indicated that all changes were significant, with the exception of help-seeking intentions.

**Table 3:** Mean scores for secondary outcome measures.

Outcome measures	T1		T2		T3	
	*n*	Mean (SD)	*n*	Mean (SD)	*n*	Mean (SD)
Depression (PHQ9)	313	5.67 (5.61)	316	5.34 (5.41)*	290	5.92 (6.03)
Quality of Life (CHU9D)	310	15.50 (6.50)	320	15.43 (6.49)	292	15.52 (7.43)
Help-Seeking (GHSQ)	313	22.08 (8.31)	308	23.12 (9.24)	266	23.25 (9.78)***
Hopelessness (Brief-Hop-Pos)	313	4.00 (1.81)	304	3.69 (1.78)**	266	3.70 (1.89)***
Suicide Literacy (LOSS)	301	6.25 (1.18)	308	6.44 (1.24)**	285	6.40 (1.35)***
Suicide Ideation (SIDAS)	-	-	315	5.92 (7.00)	286	5.94 (7.23)

Asterix indicate statistically significant changes in scores, relative to T1.

* *p* < .05;

** *p* < .01;

*** *p* < .001.

Comparing baseline to T3, hopelessness scores remained lower (4.00 vs. 3.70) and suicide literacy scores remained higher (6.25 vs. 6.40), suggesting sustained changes. Average scores for help-seeking intentions also rose at T3 relative to baseline (22.08 vs. 23.25). Paired t-tests indicated all changes were significant. Average depression scores rose slightly (5.67 vs. 5.92), although this change was not statistically significant.

Regarding the single item measure of suicide ideation, the proportion of participants indicating thoughts of suicide in the last month remained relatively stable across all three timepoints (8.9%, 7.6%, 8.2% respectively). Suicide ideation scores on the SIDAS were also similar between T2 and T3, and were generally low (5.92 and 5.94 respectively).

##### 3.3.4.2 Reframe IT-UK

Table 4 presents the mean scores for each of the secondary outcome measures for Reframe IT-UK participants. Average suicide ideation scores on the SIDAS significantly decreased from 21.90 at T2 to 15.11 after completing Reframe IT-UK (T3), which is below the SIDAS threshold of suicide risk (≥ 21). Quality of life also significantly improved, with reported difficulties falling to 22.10 at T3 (T2 = 27.90). While depression scores fell after Reframe IT-UK (T3) to 14.25 (vs. T1 = 19.43, T2 = 17.00), as did hopelessness scores, decreasing to 5.56 (vs. T1 = 6.25, T2 = 5.90), these changes were not statistically significant.

**Table 4:** Mean scores for secondary outcome measures for reframe IT-UK students.

Outcome measures	T1		T2		T3	
	*n*	Mean (SD)	*n*	Mean (SD)	*n*	Mean (SD)
PHQ9	7	19.43 (2.70)	9	17.00 (4.00)	10	14.25 (4.77)
CHU9D	8	27.88 (3.94)	10	27.90 (6.23)	10	22.10 (6.52)**
GHSQ	8	21.25 (7.38)	10	24.50 (7.18)	10	21.40 (13.83)
Brief-Hop-Pos	8	6.25 (1.98)	10	5.90 (1.60)	10	5.56 (2.56)
LOSS	9	6.13 (1.13)	9	6.22 (1.20)	10	5.00 (1.34)
SIDAS	–	–	10	21.90 (9.47)	10	15.11 (11.50)***

Asterix indicate statistically significant changes in scores, relative to T2.

* *p* < .05;

** *p* < .01;

*** *p* < .001.

### 3.4 Qualitative findings

Thematic framework analysis of qualitative feedback from young people (YP), safeTALK facilitators (F), and school staff (SS), generated two overarching themes: Benefits of Suicide Prevention Programmes in Schools, and Barriers and Facilitators to Delivery. These themes were identified consistently across all participant groups, with contributions reflecting a broad range of perspectives and experiences. Overarching findings demonstrated a strong perceived need for suicide prevention efforts in schools, with MAPSS helping to increase awareness among CYP and staff, and identify CYP who were at-risk that schools had not previously been aware of. Attitudes to the MAPSS components were mixed, with interactivity and facilitator relatability considered to be key factors influencing students' buy-in. There were also some logistical issues which presented some challenges to delivery. Thus, potential areas for future adaptations include improving the delivery and interactivity of the workshop, so that it is engaging for CYP, and advanced and careful planning of the workshops, so that the required resources are available (e.g., rooms, wi-fi, media) and they are adequately staffed. Updating the scenarios in Reframe IT-UK may also help make them more relatable for CYP.

#### 3.4.1 Benefits of suicide prevention programmes in schools

##### 3.4.1.1 Identification of young people struggling

Noted benefits included the identification of students at-risk of suicidal thoughts, including those students already known to struggle with their mental health: “definitely helpful for the ones that we are already aware have some mental health struggles” (SS4), as well as those individuals who the school had not previously been aware of: “it's identified students that we didn't know had a problem… they could have acted upon the intentions, and we wouldn't have known anything about it, so it's been brilliant.” (SS1). Parents also welcomed the information and the opportunity to seek support for their child: “obviously parents were really shocked that their child has been identified as possible high-risk. It's a concern, but they're also like ‘thank goodness you did this and that the school was part of this.’” (SS3).

##### 3.4.1.2 Increased awareness of suicide and opening up the conversation

Overall, participants felt MAPSS “opened up the conversation” about suicide and voiced a perceived need for a suicide prevention programme such as MAPSS. The nature of the workshops was thought to provide knowledge to both students and staff on how to talk about suicide in a safe and effective way:

“I think having both students and staff on the same page, working from the same framework is incredible. To be able to have a student go, ‘I know exactly what to do in this situation. I know what teacher to take you to in the situation’, that would save a life, and that is what is so important… The teachers are also equipped with the skills they need to be able to deal with that situation as well. So, I think the confidence in each other, and that bond is absolutely incredible.” (F4)

In terms of safeTALK, CYP valued the knowledge of signposting resources: “you can see all the options available for your situation” (YP11), “[safeTalk] made me aware of some helplines and stuff that I wasn't aware of” (YP12). They also felt safeTALK enhanced their ability to have conversations about suicide:

“Some of the steps they gave you when you are talking to someone, you’d use that. Before, you didn’t know how to talk to someone if they’re thinking about that kind of stuff… so it works now we know what to say” (YP6).

##### 3.4.1.3 The benefits of indicated support

Students who took part in Reframe IT-UK were generally positive, commenting that it helped to “normalise” their suicidal thoughts:

“She [the host character] really sort of comforted. Everything that she said and facts and stuff like that, and it just helped me to put myself back into like, the seat where I know that a lot of people go through this, and I'm not alone in it.” (YP1)

The mood diaries and timetabling activities were also commented on positively:

“I think mood diaries…was absolutely perfect. Because I could go back to how I was feeling, and add to it…And then I could keep track of everything that’s happening rather than just like thinking about it.” (YP3)

“I liked the video logs and the activities… one of the activities was making like a schedule. And I have a huge procrastination problem… That really helped me, because that sort of gave me some motivation to make myself a schedule and stuff like that so that really helped a lot.” (YP5)

However. students also noted some ways Reframe IT-UK could be improved moving forward, offering suggestions to include more relatable situations such as bullying or family members passing away.

##### 3.4.1.4 Training for staff

Participants welcomed the training they received and felt that it supported staff in having “difficult” conversations with students: “there was a couple of people who hadn't had any suicide intervention training whatsoever. So yeah, they found that really useful… And it opened doors for us in the future to have conversations and offer better support.” (SS4).

#### 3.4.2 Barriers and facilitators to delivery

##### 3.4.2.1 Logistical challenges of the school environment

One of the main challenges noted by staff related to the issue of securing space, particularly for the safeTALK workshops and screening: “getting two classrooms for whole mornings was tricky…I would say that was the only problematic thing we had” (SS1). Ensuring staff were able to facilitate was also an issue due to busy teaching schedules and often poor staffing levels, particularly for the “long” SafeTALK workshops: “I gave up most of my free time to sit in the room… I asked favours of teachers for all this… we don't really have the time or appropriate staffing to do it really” (SS3). School staff also noted the barriers they encountered when facilitating Reframe IT-UK. This was discussed in relation to school's IT security measures: “we couldn't get it on, our server was blocking it” (SS5). As such, staff often noted ways to address some of the solutions future participating schools could implement, such as planning in advance to ensure appropriate staffing levels (SS1) and ongoing communication between the school, MAPSS team, and the facilitators (SS4).

##### 3.4.2.2 Young people's engagement in safeTALK

The majority of participants felt the students engaged well with safeTALK: “they were brilliant. They were attentive… And so, no, there were no student challenges” (SS2). Many also spoke about what helped with engagement, such as “taking lots of little breaks” (F1) and making the session “fun”, engaging, and age-appropriate:

“We just took in loads of sweets with us…it got them talking and then you look like a bit of an idiot running around the room throwing sweets…But it just needed a bit of fun.” (F3)

Indeed, most of the comments revolved around the facilitators and their relatability:

“They've all been brilliant and been really knowledgeable and been able to answer the kids in a split second…And I think the kids were really pleased with them too, because I think they felt like they could ask any questions if they were unsure.” (SS1)

“[Trainer] has some marks around her arm from self-harm when she was at school, and she chose to wear a short sleeve top, and I thought that was really important. And one of the children did mention it.” (F1)

However, some students discussed how the workshops were “long” and “dragged”, resulting in a lack of concentration and disengagement: “after three hours you’re sort of like fidgety and bored” (YP3). Others felt that the safeTALK workshops were too didactic and “serious”: “be more, like, interactive…I think I did one group activity in the whole three hours. No one was really interacting with it because the questions were quite like, serious and harsh” (YP2).

##### 3.4.2.3 Concern over discussions about suicide

A potential school-level barrier discussed by the staff was dealing with some concern expressed by young people over parents finding out they felt suicidal after the screening: “a couple of them [students] were a little bit worried that it wasn't anonymous. They were like ‘oh will my mum find out’” (SS1). This was mirrored by the students:

“I was like, quite apprehensive to answer it [the questionnaire]. Because, if you come up as, like, quote unquote, ‘high risk’, then your parents are called and stuff. Some people can’t let their parents get told, because there will be this whole crazy thing about it..” (YP9)

Finally, while there were some concerns that parents may not want their child engaging in MAPSS, the anticipated resistance did not materialise: “I think sometimes if parents aren't fully informed… they say ‘well, my son doesn't need to know about suicide’, sometimes that can be a barrier. But we didn't have any of that” (SS4).

## 4 Discussion

The current pilot aimed to assess the feasibility and acceptability of the MAPSS programme for UK schools. Specifically, we aimed to test the acceptability and social validity of each of the MAPSS components, whether MAPSS was safe for use in UK schools, and whether a large-scale trial of MAPSS would be possible (including if we could collect enough data, if MAPSS would have the potential to be effective, and if MAPSS could be delivered as intended).

Regarding acceptability, quantitative findings suggested that safeTALK was generally considered to be appropriate for CYP. However, both quantitative and qualitative data suggests that opinions varied considerably between the two schools, with the perceived relatability of the facilitators and the interactivity of the workshops being reported broadly positively in one school, and mostly negatively in the other. This was also reflected in acceptability scores between the two schools. While the workshop's content is standardised, different organisations delivered the workshops in two schools, and it appears that the facilitator's engagement and style significantly influenced participants' perspectives of the workshop's utility. Thus, the mode of delivery is a particularly important factor influencing students' buy-in, and ultimately the acceptability of the programme. Additionally, there were issues flagged by students across both schools regarding the length of the workshops, with school staff also finding it difficult to arrange cover for that length of time and to fit it into the school timetable. This is a common issue in other school-based trials, with competing demands limiting schools' ability to take part in all required elements of a study (42).

In terms of workshop outcomes, quantitative analyses indicated significant improvements in depression, hopelessness, and suicide literacy scores after the workshop, with findings sustained at follow-up for hopelessness and suicide literacy. Participants also qualitatively reported increased knowledge around suicide. Thus, similarly to other universal school suicide prevention programmes (11–13, 17, 43), findings suggest that safeTALK has the potential to be an effective suicide awareness programme for CYP, and could be included in a larger trial. However, further work would first be needed to improve its acceptability and standardise its delivery, ensuring that content is presented in an interactive, age-appropriate, and engaging manner, with the workshops potentially condensed so they are less burdensome for both students and school logistics.

Improved suicide literacy has previously been theorised to benefit both students' own mental health and also their ability to support their peers (7), and thus workshops to promote this may be beneficial in both the immediate and long-term (e.g. (44). Improving suicide literacy may also promote help-seeking in CYP, as they are developing an understanding of the signs and symptoms of suicidal thoughts/behaviours, as well as knowledge of coping strategies and sources of support. Indeed, improved help-seeking intentions have been observed in other school-based trials (45). This will not only benefit the CYP themselves, but will also help them to support peers in the future, given that CYP are more likely to turn to their friends for support when in crisis, rather than professionals or other adults (46). Within the present study, while help-seeking scores did not change significantly immediately after safeTALK, mean scores did rise, and there was a significant increase at follow-up, suggesting that safeTALK may help to promote sustained help-seeking intentions.

Regarding Reframe IT-UK, 15 CYP were recruited onto the programme, which is approximately half of the number of CYP who were eligible to take part. This suggests that there may have been some issues with recruitment. As recruitment for Reframe IT-UK was largely the responsibility of the schools, the extent to which it was promoted or how it was advertised to CYP and their parents is not known. This needs further consideration before a larger trial, to understand how Reframe IT-UK was presented as an option to CYP and parents (particularly for those who declined participation), and to develop guidance for schools around the information that is important to pass on. However, for the CYP who did take part, acceptability was generally average, with most participants indicating that they neither agreed nor disagreed that the programme was useful. Despite this, the majority stated that they would recommend it to a friend, and none reported finding it distressing.

Changes on outcome measures for Reframe IT-UK were also promising for a future trial, with decreases in mean levels of suicide ideation, depression, and hopelessness. Findings mirror pilot work conducted of Reframe-IT in Australia (5, 18) and, taken together, suggest that, despite concerns that have been expressed regarding talking about suicide online, Reframe IT-UK has the potential to be a safe and acceptable Internet-based platform for suicide prevention activities and could be included in a larger trial. However, while suicide ideation scores fell below the identified threshold for risk after Reframe IT-UK, SIDAS scores were still higher at post-test for Reframe IT-UK participants than for the overall sample, indicating that while Reframe IT-UK may have been beneficial to the students who took part, it may be best being implemented alongside additional, more intensive support.

Furthermore, there were also some issues with engagement with relation to Reframe IT-UK, with only three students completing all eight of the modules. Qualitatively, some issues were raised which may explain this, largely relating to the logistical challenges of the school environment, including staff having the time and space to implement the sessions regularly, and the IT support that is required. Thus, further work is needed before a larger trial of MAPSS to consider the pressures and contextual constraints within the school environment, and identify ways in which Reframe IT-UK implementation can be incorporated more effectively into the school day. Given students, qualitative feedback about the “video diaries” not being relatable, further work may also be needed up update and modernise the scenarios in collaboration with CYP. Indeed, the initial scenarios were developed over a decade ago and may no longer be relevant to CYP's lives today.

In addition, qualitative findings regarding the screening component were positive, with staff consistently indicating that it had helped them to identify CYP experiencing difficulties who they were not previously aware of. This is consistent with existing evidence in the field (1, 13). While some CYP were cautious of this, largely due to anonymity, most did complete it. There was also an indication from CYP that while it was somewhat intimidating at first, they recognised that it served an important purpose. Indeed, CYP commonly have some concerns about completing mental health and wellbeing measures as part of school-based trials (47).

Finally, additional aims of the study were to assess the safety of MAPSS and the feasibility of a larger trial in UK schools. No adverse events were recorded over the course of the study and the majority of participants reported that MAPSS was not upsetting, thus suggesting it is safe to implement. Changes in scores were identified on outcome measures (as noted above), and levels of missing data were within acceptable limits for school-based research (15% T2, 30% T3), where attrition is often problematic (42). Furthermore, despite logistical issues, the majority of students did participate in safeTALK and the risk screening, suggesting that schools found an adequate solution. Additionally, 12 of the 15 young people eligible for Reframe IT-UK completed over half of the required modules, and the majority also demonstrated some additional engagement with the optional programme elements (e.g., mood diaries and message boards).

Given the rising rates of suicidality in CYP (48), and lack of appropriate or available support services both within and outside of schools, the current findings show that school-based prevention activities are promising. The multi-modal approach appears to be particularly valuable, with universal training, identification of students at-risk through screening, and indicated intervention for students at-risk each providing unique but complementary benefits. This is particularly pertinent given the UK's Government's recent “Transforming Children and Young People's Mental Health Provision” Green Paper (49); while this stated that they would be funding Mental Health Teams in schools, there was less of a focus on whole-school and multi-modal approaches to improving mental health.

### 4.1 Limitations and future directions

While the current study provides novel insights into the feasibility and acceptability of the MAPSS programme for delivery in the UK, there are limitations to consider and priorities for future trials. Firstly, as a pilot study there is a small sample size which may limit the generalisability of any findings. Participants were also all recruited from one county in England, and so experiences may not be transferable to schools and services elsewhere. Randomised trials with larger samples are needed. Secondly, the study also had some missing data, particularly for the students who participated in the Reframe IT-UK component, which limits the reliability of results and ability to detect significant statistical differences. Thirdly, only approximately half of the students eligible for Reframe IT-UK participated in the programme; further work is needed to understand why this was the case, and how uptake can be improved in the future. Qualitative interviews with the CYP who declined to participate may have been beneficial here and should be included in a future feasibility study. Fourthly, further assessments of the validity and reliability of measures is needed (particularly the SIDAS which had low internal consistency) to better determine the appropriateness and accuracy of the suite of selected measures for use in the UK. Fifth, more work is needed to adapt MAPSS in collaboration with CYP and school staff, to ensure it is acceptability and feasible to deliver in the UK.

## 5 Conclusion

The current study aimed to explore the feasibility and acceptability of a school-based suicide prevention programme in the UK, and to assess the potential for a future trial. The study indicates that MAPSS was generally acceptable for use and may be beneficial in improving suicide literacy and decreasing suicidal ideation, depression, and hopelessness in CYP. Participants also articulated a clear and urgent need for greater emphasis on school-based suicide prevention. However, attitudes to MAPSS and engagement were mixed, with the interactivity of the workshops, and relatability of the facilitators and Reframe IT-UK scenarios, considered to be key factors influencing students' buy-in. Logistical issues also presented some challenges to delivery with both the workshops and Reframe IT-UK, which in some cases precluded students’ engagement. Thus, further work is needed to refine and adapt the intervention in collaboration with CYP before future research can take place.

## Data Availability

The raw data supporting the conclusions of this article will be made available by the authors, without undue reservation.
