# Supplementary material for: Multi-modal approach to preventing suicide in schools: a regionally-based UK pilot study
Source: Front Child Adolesc Psychiatry. 2026 Mar 5;5:1768052. doi: 10.3389/frcha.2026.1768052 (PMC12999857; doi:10.3389/frcha.2026.1768052)
Supplement: Supplementary file 1 [file Table1.docx]

**Supplementary**

| **Supplementary table 1**  *Participant opinions of individual Reframe IT-UK elements* | | | | |
| --- | --- | --- | --- | --- |
| **Reframe IT-UK Element** | **Disliked** | **Neither Liked or Disliked** | **Liked** | **N/A (Did Not Use) or Missing** |
| Video diaries | 1 | 5 | 0 | 4 |
| CBT activities | 2 | 1 | 3 | 4 |
| Overall look and feel | 0 | 3 | 3 | 4 |
| Message board | 0 | 2 | 3 | 6 |
| Topics covered | 1 | 2 | 3 | 4 |
| Host character | 1 | 2 | 2 | 5 |
| Female character | 1 | 2 | 2 | 5 |
| Male character | 1 | 2 | 2 | 5 |
|  | **Unhelpful** | **Neither helpful or unhelpful** | **Helpful** | **N/A (Did Not Use) or Missing** |
| Recognising my emotions | 2 | 2 | 3 | 3 |
| Identifying my 'problem situations' | 1 | 2 | 4 | 3 |
| Identifying my 'tipping point' | 1 | 3 | 3 | 3 |
| Recognising my 'unhelpful thoughts' | 1 | 1 | 5 | 3 |
| Learning who I can go to for help | 1 | 3 | 3 | 3 |
| Scheduling activities I enjoy | 2 | 3 | 2 | 3 |
| Problem solving | 1 | 3 | 2 | 4 |
| Replacing unhelpful thoughts with helpful thoughts | 2 | 3 | 1 | 3 |


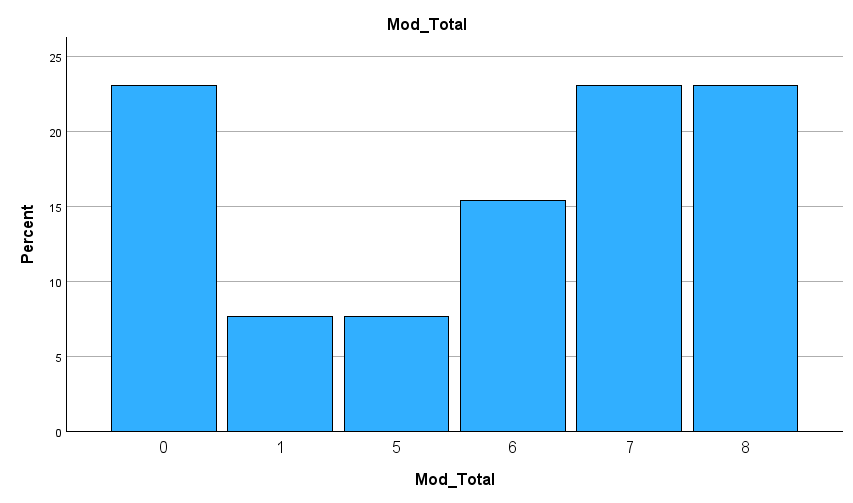


**Supplementary Figure 1. Bar chart of the percentage of modules completed.**

**Number of Modules**


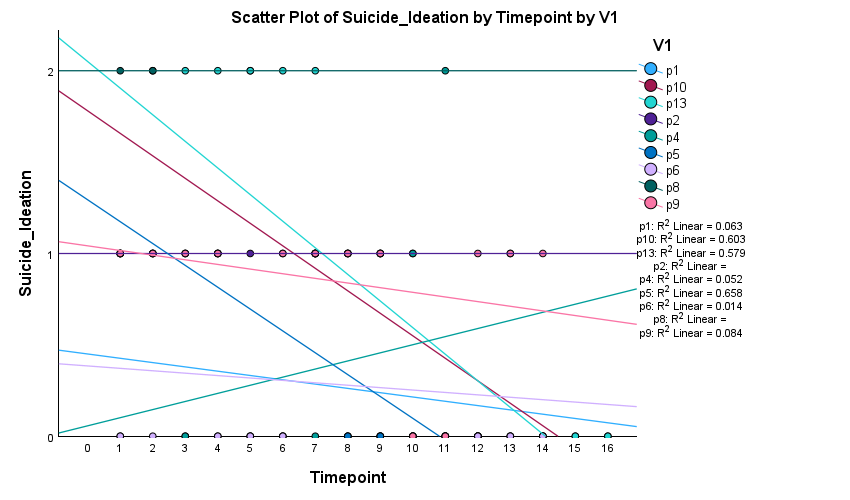


**Figure 2. Scatterplot of suicide ideation by timepoint for each participant.**

**Participant**
